# Genetics and Epigenetics of Bone Remodeling and Metabolic Bone Diseases

**DOI:** 10.3390/ijms23031500

**Published:** 2022-01-28

**Authors:** Lucia Oton-Gonzalez, Chiara Mazziotta, Maria Rosa Iaquinta, Elisa Mazzoni, Riccardo Nocini, Lorenzo Trevisiol, Antonio D’Agostino, Mauro Tognon, John Charles Rotondo, Fernanda Martini

**Affiliations:** 1 Department of Medical Sciences, University of Ferrara, 64/b, Fossato di Mortara Street, 44121 Ferrara, Italy; lucia.otongonzalez@unife.it (L.O.-G.); chiara.mazziotta@unife.it (C.M.); mariarosa.iaquinta@unife.it (M.R.I.); tgm@unife.it (M.T.); 2 Center for Studies on Gender Medicine, Department of Medical Sciences, University of Ferrara, 44121 Ferrara, Italy; 3 Department of Chemical, Pharmaceutical and Agricultural Sciences, University of Ferrara, 44121 Ferrara, Italy; elisa.mazzoni@unife.it; 4 Unit of Otolaryngology, University of Verona, 37134 Verona, Italy; riccardo.nocini@univr.it; 5 Unit of Maxillo-Facial Surgery and Dentistry, University of Verona, 37134 Verona, Italy; lorenzo.trevisiol@univr.it (L.T.); antonio.dagostino@univr.it (A.D.); 6 Laboratory for Technologies of Advanced Therapies (LTTA), University of Ferrara, 44121 Ferrara, Italy

**Keywords:** osteogenesis, bone formation, bone remodeling, bone morphogenic protein, DNA methylation, histone post-translational modifications, non-coding RNA, microRNA, long non-coding RNA, metabolic bone disease, bone disease

## Abstract

Bone metabolism consists of a balance between bone formation and bone resorption, which is mediated by osteoblast and osteoclast activity, respectively. In order to ensure bone plasticity, the bone remodeling process needs to function properly. Mesenchymal stem cells differentiate into the osteoblast lineage by activating different signaling pathways, including transforming growth factor β (TGF-β)/bone morphogenic protein (BMP) and the Wingless/Int-1 (Wnt)/β-catenin pathways. Recent data indicate that bone remodeling processes are also epigenetically regulated by DNA methylation, histone post-translational modifications, and non-coding RNA expressions, such as micro-RNAs, long non-coding RNAs, and circular RNAs. Mutations and dysfunctions in pathways regulating the osteoblast differentiation might influence the bone remodeling process, ultimately leading to a large variety of metabolic bone diseases. In this review, we aim to summarize and describe the genetics and epigenetics of the bone remodeling process. Moreover, the current findings behind the genetics of metabolic bone diseases are also reported.

## 1. Introduction: Bone Structure and Cell Types 

Bone is a living tissue that supports and protects several organs in the body and provides the environment for blood cell production. It is a metabolically active tissue that undergoes a constant cycle of resorption and replacement. This continuous process allows bone to adapt to the changes required for healthy functioning, to maintain bone strength, and the changes required for fracture healing to take place [1,2,3]. Furthermore, bone tissue plays an important role in mineral homeostasis, such as calcium and phosphorus, and gives a solid base for skeletal muscles [4]. 

Normal bone tissue consists of two phases, i.e., an organic and an inorganic phase. In the organic phase, osteoblasts and osteoclasts are the major components of bone tissue cells, underpinning the main metabolic activities in bone. Osteoblasts, which are cells responsible for bone matrix synthesis, derive from mesenchymal stem cells (MSCs) in bone marrow [5], blood, and pericytes [6,7]. MSC migration is a complex mechanism, which is significantly important for both bone formation and fracture healing. Nevertheless, its regulation system is yet to be elucidated [6,8,9]. Alterations in MSC migration can lead to abnormal bone imbalances [7,10]. An equally important role in bone homeostasis is attributed to osteoclasts, which are multinucleated cells derived from progenitors of the monocyte/macrophage lineage that digest the components of bone allowing for bone resorption and replacement [11,12]. Other cell types that reside in bone tissue are bone lining cells and osteocytes. Specifically, the first is osteoblast-derived cells, which cover all quiescent bone surfaces where bone resorption or bone formation are not requested [13]. Conversely, osteocytes derive from osteoblasts that suspend their activity when buried in the matrix, acting as mechano-sensors capable of transducing mechanical strengths into biological signals [14]. When combined, these cells are organized into temporary anatomical structures named basic multicellular units (BMUs). BMUs carry out bone remodeling, a biological process that results in structural changes and skeletal renewal [5]. 

Bone matrix is considered to form the intercellular substance of bone tissue and the inorganic phase. The extracellular matrix (ECM) consists primarily of type I collagen (COL1), which is the most abundant protein in bone tissue, complexed with a crystalline inorganic component composed of hydroxylapatite (HA; Ca_10_(PO_4_)_6_(OH)_2_) with citrate, carbonate, and ions, such as F^−^, K^+^, Sr^2+^, Pb^2+^, Zn^2+^, Cu^2+^, Mg^2+^, and Fe^2+^ [15,16,17]. The mechanical quality of the bone matrix is influenced by non-collagenous proteins [18]. The principal non-collagenous proteins of the bone matrix are bone sialoprotein (BSP), osteonectin (ON), osteopontin (OPN), and osteocalcin (OCN) [19] which contain aspartic acid (Asp) and glutamic acid (Glu) residues, with high affinity for calcium ions (Ca^2+^) due to their charged carboxyl groups [19]. In BSP, polyglutamic acid segments are responsible for binding the protein to apatite, while the same role in OPN is operated by polyaspartic acid segments [20]. Studies on OPN reported that it behaves like bone glue [18,20]. ON is a cysteine amino acid-rich protein that is expressed in mineralized tissues. ON is also involved in osteoblast differentiation and osteoclast activity [21]. OCN, which is expressed by osteoblasts, is also known as bone g-carboxyglutamate protein (BGLAP) and is frequently used as a clinical marker of bone turnover [22]. Interestingly, different amounts of non-collagenous bone protein are contained in bone types. For example, cortical bone contains 30× more OCN than trabecular bone, while the ON excess in trabecular bone ranges from 21- to 47-fold [23]. 

Two main histological types of mature bone can be identified, (i) cortical or compact bone, which represents 80% of bone mass; it presents a dense and ordered structure, (ii) cancellous or trabecular bone, which is lighter and less compact than the cortical bone [24,25]. In addition, cortical or compact bone is located mainly on the surfaces of flat bones and in the shaft of long bones. It is made of bone laid down concentrically around central canals, known as Haversian systems. Blood vessels, lymphatics, nerves, and connective tissue are contained in these structures [25]. Trabecular or cancellous bone, on the other hand, has an irregular structure [25]. It is composed of a honeycomb-like network of trabecular plates, forming the ends of long bones and the central parts of flat bones [26]. 

## 2. Pathways Involved in Bone Metabolism

**Figure 1:**
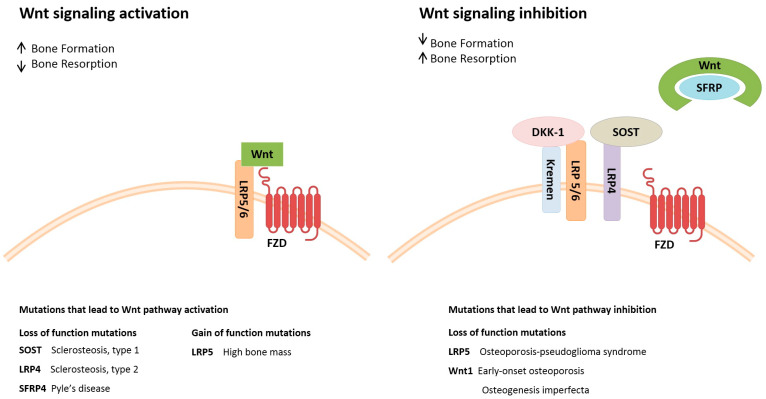
Schematic illustration of the Wingless/Int-1 (Wnt) signaling pathway. The Wnt signaling pathway and its components reported to be mutated in metabolic bone disease are reported. Activation of the canonical Wnt pathway leads to an increase in bone mass. Wnt ligands interact with co-receptor LRP5/6 and frizzled (FZD) to activate the Wnt signaling pathway. The inhibition of Wnt signaling is mediated by extracellular factors, such as sclerostin (SOST) and Dickkopf-related protein 1 (DKK-1) and leads to bone mass decrease. DKK-1 binds to the LRP5/6 co-receptor, thereby preventing activation by Wnt ligands. Inhibitory transmembrane protein LRP4, which is a SOST-interacting protein, is recruited and the Kremen proteins, which are high-affinity DKK-1 receptors, cooperate with DKK-1 to decrease Wnt signaling. In addition, secreted frizzled-related protein (SFRP) inhibits the canonical Wnt pathway by sequestering Wnt ligands. Mutations in Wnt signaling components result in pathway activation/inhibition. For example, loss-of-function mutations affecting SOST and LRP4 lead to Wnt pathway activation causing bone tissue sclerosteosis, as well as loss-of-function mutations of LRP5 and the WNT1 ligand result in Wnt signaling pathway inhibition leading to osteoporosis disorders.

Bone metabolism consists of coupled and dynamic processes orchestrated by both osteoblasts and osteoclasts. In order to ensure bone plasticity, the bone remodeling process needs to function properly [27]. Osteoblasts derive from MSCs that differentiate into the osteoblast lineage by activating different signaling pathways, the main ones being (i) transforming growth factor β (TGF-β)/bone morphogenic protein (BMP) and (ii) Wingless/Int-1 (Wnt)/β-catenin pathways (Figure 1). 

However, other regulators of the ostegenic process are Hedgehog (Hh) and NOTCH cascades, Sox9 and Msx2 genes, Histone deacetylases (HDACs), fibroblast growth factor (FGF), and parathyroid hormone-related peptide (PTHrP) cytokines [28,29]. These molecular pathways and regulators cooperate in order to induce the expression of the main osteogenic transcription factors, such as Runt-related transcription factor 2 (Runx2) and Sp7/Osterix (Osx). Runx2 activates OCN, OPN, ON, and COL1 expression leading to osteogenesis [3,6]. Mature osteoblasts also directly regulate osteoclastogenesis and control Ca^2+^ released by osteoclasts, thus impacting Ca^2+^ levels in the blood [30]. 

Osteoclast activity is regulated by different factors and signaling cascades, such as BMPs, calcitonin, interleukins 1/6/11, PTHrP, Wnt cascade, receptor activator of nuclear factor kappa-B (NF-κβ) ligand (RANKL), and macrophage colony-stimulating factor (M-CSF) [31]. Interaction between M-CSF and its receptor c-Fms induces osteoclast proliferation by activating Src, PLC-, PI(3)K, Akt, and Erk kinases. In addition, RANKL binds to its receptor, named Receptor activator of NF-κB (RANK), and promotes the association of RANK to TRAF6, thus activating Erk, JNK, and p38 kinases. The triggered kinases positively regulate the expression of the main transcription factor for osteoclasts formation and function, named Nuclear Factor of Activated T Cells 1 calcineurin-dependent (NFATc1). Osteoprotegerin (OPG) acts as a decoy receptor for RANKL, inhibiting osteoclast activity and promoting apoptosis. Specifically, OPG binds RANKL inhibiting its interaction with RANK, thus preventing excessive bone resorption. Wnt/β-catenin pathway stimulates OPG expression leading to a reduction in osteoclast differentiation [32]. In summary, the RANK/RANKL/OPG system is a key component in bone metabolism. 

## 3. The Bone Turnover Cycle

Bone remodeling is a continuous process throughout life [10]. It consists of some sequential steps, i.e., the initiation, reversal, and termination phases [33]. During the initiation phase, bone resorption consists of recruiting osteoclast precursors, which differentiate into mature osteoclasts. Osteoclastogenesis requires specific key mediators, such as M-CSF and RANKL. Specifically, M-CSF, which is produced by osteoblasts and many other cell types, is required for osteoclast precursor proliferation, as well as their differentiation and fusion into osteoclasts [34]. Furthermore, RANKL is expressed by several types of cells, including osteoblasts [35]. RANKL binds its receptor, which is localized on the surface of osteoclast precursors allowing fusion, maturation, survival, and osteoclast activation [36]. Several studies reported that osteocytes produce the majority of the RANKL required for osteoclast formation in bone remodeling [37,38].

During bone resorption, many factors that lead to MSC recruitment and differentiation are released through bone remodeling to enable bone formation in the bone marrow microenvironment [39]. Bone resorption inhibition occurs in the subsequent transient, or reversal phase, where osteoblasts are recruited to allow bone formation. Osteoblasts may produce osteoprotegerin protein (OPG), which is a decoy receptor for RANKL, preventing its binding to RANK, with the consequent inhibition of osteoclast activation and differentiation [12].

The termination phase represents the final step of the remodeling cycle. In this phase, an equal amount of resorbed bone is replaced [40]. Osteocytes contribute to completing the remodeling process by producing sclerostin, a small protein encoded by the SOST gene, which inhibits bone formation induced by Wnt signaling in osteoblasts [33,41]. At the end of the process, mature osteoblasts undergo apoptosis, become bone lining cells or differentiate into osteocytes [5].

The fine balance between bone resorption and bone formation allows skeleton integrity to be maintained. The coordinated activity of these cells, as well as the integrity of the calcium and phosphate homoeostatic mechanisms, which are primarily mediated by the parathyroid hormone, FGF23, and vitamin D, are required for normal bone formation, metabolism, and repair [42]. Alterations to this mechanism, such as dysfunctions in pathways and factors that regulate osteoblastic bone formation and osteoclastic bone resorption may lead to the onset of bone metabolic diseases, including osteoporosis [43], which is caused by excessive bone resorption, or osteopetrosis due to inadequate osteoclast function and excessive bone formation [44,45].

## 4. Epigenetics in Bone Remodeling Processes 

Gene expression is modulated by several different mechanisms/factors [46,47,48,49], including epigenetic processes. A growing number of studies demonstrated that bone remodeling processes are epigenetically regulated by DNA methylation [50] and non-coding RNAs, such as micro-RNAs (miRNAs) [51], long non-coding RNAs (lncRNAs), and circular RNAs (circRNAs) [52].

### 4.1. DNA Methylation

DNA methylation is the process of transferring a methyl group to the 5′ position of cytosines in CpG dinucleotides in the DNA sequence [30,53]. DNA methylation occurs mainly on CpG islands in gene promoter regions [54,55]. The enzymes that catalyze DNA methylation are the DNA methyltransferases (DNMTs), including DNMT1, DNMT2, and DNMT3 [56,57]. Demethylation is the reverse process of methylation in which a methyl group is removed [58,59]. This process is catalyzed by several DNA demethylases, such as TET1, OCT4, and GADD45A, and results in hypomethylation [60,61]. Generally, the methylation status of promoter regions is inversely correlated to gene expression [62]. High methylation levels can suppress bone-related gene activation, thus impairing osteogenesis [55,60]. In contrast, low methylation levels in the promoter regions increase the expression of genes involved in osteogenic differentiation [55,60]

Both methylation and demethylation processes are implicated in bone turnover. Published studies have shown that DNA methylation in bone plays a fundamental role in controlling genes associated with both osteoblast and osteoclast differentiation, including RUNX2, OSX, OCN, ALP, Wnt, RANK/RANKL/OPG, and other important signaling pathways [61,63,64,65,66].

Methylation status can determine the fate of MSCs from various sources. Bone marrow-derived MSCs (BMSCs) tend to differentiate into osteoblasts, whereas adipose isolated MSCs (ASCs) tend to turn into adipocytes. This may be due to the hypomethylation of the promoter regions of specific osteogenic genes, such as RUNX2 and OCN, in BMSCs, whereas complete hypomethylation of the adipose tissue-related gene PPAR-g2 was observed in ASCs. In addition, the expression of the corresponding mRNAs is also higher [61]. Moreover, the RUNX2 promoter of ASCs is hypermethylated, resulting in low mRNA expression of RUNX2 [63].

RUNX2 and OSX are specific transcription factors involved in the bone formation process and osteoblast differentiation. OSX is the downstream target of RUNX2 [67,68]. RUNX2 methylation levels are reduced during osteogenic differentiation in MSCs, suggesting that RUNX2 methylation is involved in regulating osteoblast differentiation [69]. Moreover, the methylation level in the promoter region of OSX changes dynamically during osteogenic differentiation, suggesting that OSX epigenetic regulation may modulate osteogenic differentiation in MSCs. Inhibition of DNA demethylase reversed the expression levels of these genes, suggesting that RUNX2 and OSX are mainly regulated by DNA methylation mechanisms during osteogenic differentiation in ASCs [61]. 

Of all the growth factors that stimulate osteogenic differentiation in MSCs, BMP2 plays a fundamental role [70]. Hypermethylation of the BMP2 promoter region in osteoblasts leads to the silencing of certain genes associated with bone formation [71,72]. Indeed, the methylation level of the BMP2 promoter has been reported to significantly correlate with the degree of osteoporosis in affected patients, resulting in BMP2 downregulation [72].

Methylation levels are variable and can change during differentiation and in response to external stimuli [73]. Indeed, promoter hypomethylation in an early osteogenic marker, that is ALP, has been shown to induce high ALP expression in osteoblasts [64]. However, downregulation of ALP expression was found in mature osteocytes due to hypermethylation in its promoter region [64]. These results suggest that the DNA methylation pathway may inhibit the expression of ALP during osteogenic differentiation and may be time-dependent and variable during differentiation [64,74]. Moreover, OCN, an important marker of osteogenic differentiation [75], exhibited a high level of methylation of its promoter during the first differentiation phase in primary osteoblasts, which gradually decreased. These results suggest that OCN hypomethylation may promote osteogenesis [76].

The Wnt/β-catenin signaling pathway is one of the major signaling cascades involved in MSC osteoblast differentiation [77]. The genes in the Wnt signaling pathway are also epigenetically regulated by DNA methylation. During BMSC osteogenic differentiation, a decrease in methylation has been detected in the receptor tyrosine kinase-like orphan receptor 2 (ROR2) promoter region of the Wnt signaling pathway [65]. 

As mentioned above, bone remodeling is also regulated by the RANKL/RANK/OPG system [78]. DNA methylation of RANKL and its receptor OPG plays a key role in regulating osteoclast differentiation [79]. Quantitative methylation of all types of bone cells and pyrolysis sequence analysis showed that methylation of the transcription initiation regions of RANKL and OPG inhibits expression of RANKL and OPG genes [66,79,80]. Therefore, methylation regulation of RANKL/RANK/OPG plays an important role in bone regeneration [79,81].

Studies have shown that DNMTs play a fundamental role in bone homeostasis. Moreover, methylation inhibitors can affect osteogenesis [82,83]. The most important DNA methylation inhibitors include nucleoside analogs, such as 5-aza-2’-deoxycytidine (5-aza-dC) and 5-aza-C [84,85]. Among them, 5-aza-dC and 5-aza-C are the most used DNA methylation inhibitors [84,85]. MSCs treated with 5-aza-C undergo genome demethylation which promotes the expression of osteogenesis-related genes and thus osteogenic differentiation [86]. In addition, 5-aza-dC demethylates the promoters of distal-less homeobox 5 (DLX5) and OSX genes while inducing the expression of osteogenic markers, such as ALP and OCN [87]. In addition, DNMT3a has been reported to promote osteoclast differentiation and bone resorption by inhibiting interferon regulatory factor 8 (IRF8), which negatively regulates osteoclast differentiation. DNMT3a inhibits IRF8 primarily by enhancing methylation of the remote regulatory element IRF8 [88].

Finally, osteolytic changes in myeloma patients have been reported to be related to an increase in IRF8 methylation and IRF8 down-expression [89]. Inhibition of IRF8 further induces bone resorption, suggesting that epigenetics may be a potential target for bone disease treatment [89].

### 4.2. Histone Post-Translational Modifications

Histones H1, H2A, H2B, H3, and H4 are small nuclear proteins rich in positively charged basic amino acids, i.e., arginine and lysine, which can interact with the negatively charged DNA [56]. The interaction between an octamer of four histones (H2A/2B/3/4) and a 147-bp segment of DNA leads to chromatin compaction and assembly into the nucleosome, which is the basic chromatin unit [56]. The continuous nucleosome formation and folding are at the bases of the chromatin remodeling processes that regulate gene expression. Histone post-translational modifications (PTMs) occur at the *N*-terminal position of these nuclear proteins, thereby promoting changes in the chromatin structure. PTMs modulate the expression of genes, ultimately leading to the regulation of a large variety of cell functions, including osteogenesis [81,90,91]. The main histone modifications comprise (de)acetylation, (de)methylation, (de)phosphorylation, and ubiquitylation modifications [56]. Histones PTMs are modulated by several classes of modifying enzymes, including (i) histone acetyltransferases (HATs) and histone deacetylases (HDACs); (ii) histone methyltransferases and demethylases, and (iii) histone kinases and phosphatases, that promote or eradicate specific modifications, respectively [56]. 

A growing body of evidence indicates that histones PTMs and, in particular, histone (de)acetylation, (de)methylation, and modifying enzymes play a role in bone remodeling processes [81,90,91]. Two genome-wide studies characterized the chromatin landscape during MSC differentiation [92,93]. A global enrichment of a variety of histone marks has been identified as essential for multipotent differentiation of MSCs, including H4K5ac, H3K4me3, H3K9ac, H3K27ac, and H3K36me3, H3K4me1, H3K4me3 [92,93].

Histone marks and modifying enzymes localized within specific bone remodeling candidate genes have been identified. Indeed, PTMs, such as acetylation and methylation at histones associated with promoters of bone-related genes are functionally related to the chromatin remodeling processes that regulate their expression during bone remodeling [94]. 

An enrichment in the H3K4me3 and H3K27ac marks at the Runx2 promoter region has been related to the epigenetically-forced expression of RUNX2 and osteocalcin under myoblastic differentiation [95]. Moreover, H3/H4 acetylation marks have been found to enhance the promoters of the osterix and osteocalcin genes, while a reduction in histone deacetylase 1 recruitment has also been determined at those promoters [96]. The implication of histone acetylation upon the expression of osteocalcin has also been investigated in the context of osteoblastogenesis. A transcriptionally active osteocalcin gene has been linked to acetylated histones H3 and H4 localized in the osteocalcin promoter region [97,98]. Moreover, these modifications seem to be mediated by a complex interaction between different factors, including the transcriptional coactivator and HAT p300, alongside PCAF and RUNX2 [99]. Additional studies indicated that the acetylation of both H3 and H4 at the osteocalcin promoter can be prevented by NFATC1, which specifically interacts with HDAC3 [100], being capable, in turn, to antagonize the transcriptional activity of RUNX2 [101,102]. The suppressive role of HDAC3 on osteogenic differentiation by deacetylating H3 localized on bone-related genes has also been demonstrated at the bone sialoprotein promoter [103]. Previous data indicated that the HAT PCAF, which is a p300/CBP-associated factor (PCAF/KAT2B), is implicated in the osteogenic commitment of MSCs [90], while CBP and p300 have also been found nearby promoters of osteoblastic genes during osteoblast differentiation [90]. A stimulated expression of PCAF has been determined following Smad-driven osteogenic induction, accompanied by an overexpression of BMP pathway genes by H3K9 acetylation [104]. Moreover, additional HATs, such as MOZ-related factor (MORF/KAT6B) and monocytic leukemia zinc finger protein (MOZ/KAT6A), have also been found to play a role in osteogenesis by interacting with RUNX2 [105]. 

Histone methylation on the promoters of bone-remodeling-related genes also plays a role in osteogenesis. For instance, the H3K36 tri-methyltransferase Wolf–Hirschhorn syndrome candidate 1 (WHSC1 or NSD2) has been reported to interact with both RUNX2 and p300, thereby leading to H3K36 trimethylation expression of downstream bone-related genes [106,107]. Enhancer of zeste homolog 2 (EZH2/KMT6) can suppress osteoblastogenesis via H3K27me3 mark on the promoters of osteoblast-related genes [107,108]. However, an intriguing aspect of EZH2 is that this enzyme seems to play a dual role during bone formation as it seems to either favor the proliferative expansion of osteoprogenitor cells or suppress osteoblast-related genes [109]. Indeed, this enzyme can lead to osteogenic differentiation of MSCs by epigenetically regulating important osteogenic genes, such as RUNX2, MX1, ZBTB16 other than OP, OC, and FHL-1, via H3K27me3 mark on their promoters [108,110]. An additional histone methyltransferase named SUV420H2 has been found to be implicated in osteoblast differentiation. The functional silencing of SUV420H2 by a siRNA model can lead to a global decrease in H4K20 methylation alongside a reduced expression of osteogenic transcription factors and bone-related genes [111]. A recent in vitro study identified Lysine-specific demethylase 1 (LSD1), which removes H3K4/K9 mono-/di-methylation marks, as an important inhibitor of the differentiation of human MSCs toward osteoblasts [112]. In particular, the functional silencing of LSD1 osteoblast progenitor cells leads to an increased bone mass by negatively regulating the expression of BMP2 and WNT7B through H3K4me2 methylation loss on their promoters [112].

In summary, the aforementioned investigations underlined the important role of histone-modifying enzymes and histone modifications, such as acetylation and methylation, which occur on the promoters of bone-regulating genes, in the regulation of the bone remodeling process.

### 4.3. Non-Coding RNAs 

ncRNAs are RNA molecules without protein-encoding capability [3]. ncRNAs can epigenetically regulate gene expression by inhibiting the translation of their mRNA targets. ncRNA classes, involved in the regulation of bone metabolism, include miRNAs, lncRNAs, and circRNAs. 

miRNAs are small RNA transcripts consisting of ∼22 nucleotides that regulate gene expression by binding 3′-UTR of mRNA targets [113]. The complete complementarity of miRNAs with the mRNA target induces its degradation [56], while miRNA-mRNA incomplete binding leads to mRNA expression inhibition [114]. Several studies demonstrated miRNA involvement in the epigenetic regulation of bone remodeling. Specifically, miRNAs regulate bone homeostasis by inhibiting the positive or negative transcription factors implicated in the pivotal osteoblast and osteoclast differentiation pathways. miRNAs that target osteogenic factors, including Runx2, Osx, Opn, Ocn, and BMPs, are negative regulators of osteoblastic differentiation. Contrarywise, miRNAs that suppress the activity of osteogenic inhibitors, such as Smad7 and DKK-1, positively regulate osteoblast formation [28]. Similarly, miRNAs that inhibit the expression of RANK, RANKL, NFATc1, and TRAF6, suppress osteoclastogenesis [51]. Moreover, miRNAs also control the RANKL/OPG ratio, thus influencing osteoclastic differentiation [115].

lncRNAs are transcript molecules of ∼200 nucleotides that regulate gene expression by inducing chromatin modification or by inhibiting mRNAs’ target expression [116]. lncRNAs can also indirectly regulate mRNA expression by acting as a miRNA sponge. Specifically, they suppress the inhibitory activity of miRNAs and allow the expression of its mRNA target [3]. lncRNAs’ activity is implicated in bone homeostasis. Growing evidence has shown a role for lncRNAs in the bone formation process by stimulating the expression of osteogenic transcription factors. Most of these induce osteogenesis by cross-talking to miRNAs, thus blocking their inhibitor activity on osteogenic factors [3]. Despite osteoblast differentiation, the role of lncRNAs in osteoclast differentiation is still largely unknown. lncRNA AK077216 promotes osteoclast differentiation-inducing NFATc1 expression [117], whereas lncRNA-NONMMUT037835.2 negatively regulates osteoclastogenesis by inhibiting the expression of the main osteoclastic factors, including RANK and NF-κB/MAPK signaling pathways [118]. Other lncRNAs stimulate osteoclastogenesis and bone resorption by acting as a miRNAs sponge, such as lnc-MIRG, lnc-MALAT1, and lnc-Neat1, that bind miR-1897, miR-124, and miR-7, respectively [119].

circRNAs are circular transcripts with a length of hundreds or thousands of nucleotides derived from alternative splicing [120]. Due to their circular conformation, circRNAs are resistant to exonuclease digestion, including by the RNAse R enzyme [121]. circRNAs process RNA, regulate the transcription of their parental genes, and act as miRNAs sponges [122]. In recent years, accumulating epigenetics data revealed the involvement of circRNAs in bone metabolism regulation. Several bioinformatic analyses showed differentially expressed circRNAs during BMSC osteogenic differentiation [121,123], and in hematopoietic stem cells (HSCs) [124]. circRNA hsa_circ_0074834 helps BMSCs toward an osteogenesis–angiogenesis combined process, regulating the expression of osteogenic factors ZEB1 and VEGF by sponging miR-942-35p [125]. Hsa_circ_0006393 promotes osteogenic pathway activation by binding miR-145-5p, thus upregulating the forkhead box O1 (FOXO1) gene. Indeed, this circRNA is downregulated in glucocorticoid-induced osteoporosis (GIOP) [126]. Moreover, circRNA_009934 expression levels increase during osteoclastogenesis and are correlated with bone resorption. Predictive results demonstrate that this circRNA acts as a positive regulator of TRAF6 expression by blocking miR-5107 activity [127]. An in vivo experiment revealed that the osteoclast differentiation of bone marrow monocyte/macrophage (BMM) cells is positively regulated by molecular axis consisting of circRNA_28313- miR-195a- CSF1 [128]. The few studies reported herein prove how lnc-RNAs can epigenetically influence the bone remodeling process. 

## 5. Genetics of Metabolic Bone Diseases 

Metabolic bone diseases include a varied group of disorders characterized by alterations in skeletal homeostasis, which are often associated with abnormal circulating concentrations of calcium, phosphate, and/or vitamin D metabolites [129]. Metabolic bone diseases represent the third most common endocrine disorders after thyroid diseases and diabetes [130]. 

Excluding osteoporosis, which is considered the most common of these, bone metabolic diseases comprise a large group of different disorders with low prevalence in the general population. Among these disorders, the most common are Rickets, Osteomalacia, and Juvenile Paget’s disease. Osteoporosis affects approximately 200 million individuals worldwide, while it has been estimated that about 1 in 3 and 1 in 5 females and males, respectively, exhibit low bone mass and/or osteoporotic fractures during their lifetime [131]. Variable Rickets rates have been reported according to different geographical areas, being reported as merely sporadic in some areas, such as Europe [132], while in other areas, e.g., low-income countries, up to 9% of the childhood population is affected [133]. The prevalence of osteomalacia and Juvenile Paget’s disease has been estimated as ranging between 1–5% and 1–2% of the general population, respectively [134,135].

Commonly, these diseases present a genetic basis and represent either a (i) monogenic disorder due to a germline or somatic single-gene mutation, or a (ii) digenic, oligogenic, and polygenic disorder that involves variants in more than one gene [136]. Inheritance patterns of monogenic disorders occur as one of the following traits: (i) autosomal dominant, (ii) autosomal recessive, (iii) X-linked dominant and recessive, (iv) Y-linked, (v) non-Mendelian mitochondrial defects. Moreover, monogenic metabolic bone diseases may also be caused by sporadic postzygotic mosaicism. Germline single-gene mutations causing Mendelian diseases typically have high penetrance, whereas the genetic variations causing oligogenic or polygenic disorders are each associated with smaller effects with additional contributions from environmental factors (multifactor diseases) [129]. Notably, the same metabolic bone disease usually presents multiple modes of genetic inheritance. Therefore, the same disease can present different genetic backgrounds, which convergently leads to the same phenotype. As a result of different mutations and dysfunctions in pathways that regulate bone turnover, several metabolic bone diseases can be described, some of them are outlined below.

### 5.1. Osteoporosis

Osteoporosis is the most common metabolic bone disease. This disease is mainly characterized by reduced bone mineral density with the consequent deterioration of bone tissue and its microarchitecture [137]. This leads to bone fragility with an increased risk of fractures, particularly to the wrist, spine, and hip, the latter being associated with a 12–24% and 25% mortality rate, within the first year of fracture, in females and males, respectively [138]. These statistics are predicted to increase as the result of global aging in the world population. Osteoporosis can be associated with complicated polygenic characteristics, with more than 200 loci linked to it, or as a monogenic condition [129,139]. Indeed, genetic factors play an important role in the development of osteoporosis. 

Mutations in more than 15 genes with both structural and regulatory functions have been implicated in the pathogenesis of osteoporosis. These genes largely comprise regulators of bone metabolism, including local regulators of bone metabolism and bone matrix components, as well as cell receptors and calciotrophic hormones [140]. Among them, Vitamin (1, 25-dihydroxyvitamin) D receptor (VDR), estrogen, and androgen receptors and the Collagen type I α (COLIA1) gene have been the most extensively investigated; both polymorphisms and mutations within the regulatory and coding sequences of these genes have been identified as related to osteoporosis [140].

The VDR gene is located on chromosome 12q12-q14 and encodes for the vitamin D receptor [141]. VDR plays a key role in bone metabolism and maintenance of serum calcium homeostasis by binding to its ligand vitamin D and regulating the expression of the response genes. Morrison et al., in 1994, first identified polymorphisms in the 3′ region of the VDR, which have been linked to low ostecalcin levels and an increased risk of osteoporotic fracture [142]. Overall, allelic variation of this gene has been related to up to 75% of the genetic effect on bone mineral density. However, the relationship between VDR-3′ genotype and bone mineral density may be modulated by high vitamin D and calcium intake. VDR-5′ polymorphism has also been identified and related to low bone mineral density in elderly individuals and intestinal calcium absorption in children [143,144].

Both estrogen and androgen receptors play a critical role in regulating bone growth and in maintaining skeletal mass [145,146]. Two isoforms of the human estrogen receptor, i.e., estrogen receptor alpha (ERα) and estrogen receptor beta (ERβ), are expressed by two different genes named ESR1 and ESR2, which are located at chromosome 6p25.1 and 14q22-q24, respectively [147]. In particular, ESR1 is considered a strong candidate gene for osteoporosis [147]. Previous studies indicated that both polymorphisms and mutations at ESR1 play an important role in the onset and development of this metabolic bone disease. A TA dinucleotide repeat in the ESR1 promoter and are two single nucleotide polymorphisms in the first intron give rise to reduced bone mineral density in postmenopausal and premenopausal women, while potentially related to the acquisition of peak bone mass [148]. However, the mechanisms behind these polymorphisms and osteoporosis is still unclear. Regarding the androgen receptor, a polymorphic (AGC)n repeat trinucleotide polymorphism, which has been identified in the first exon of the coding sequence, has been associated with reduced transcriptional activity of the receptor, thereby leading to a reduced bone mass in women with high levels of sex hormone-binding globulin (SHBG) and with an increased risk of osteoporosis fractures [147].

Mutations in NR3C1, a glucocorticoid receptor (GC), altered the expression of ECM-, osteoblast-, and osteoclast-related genes [149]. The NR3C1 BclI gene C/G polymorphism has been proposed to be one of the genetic causes of osteoporosis [150]. Furthermore, GC receptor gene polymorphism was previously shown to be closely related to bone mineral density (BMD). The haploid frequency of SNP rs1866388 was higher in patients with higher BMD. In addition, the SNPs, rs1866388 and rs2918419, differed between individuals with extremely low and high BMD and seemed to be involved in BMD regulation in a gender-dependent manner [151].

COLIA1 gene is located on chromosome 17q21.31-q22 and encodes the alpha I chain of type 1 collagen. This gene is particularly considered as a candidate for susceptibility to osteoporosis since type I collagen is the major structural bone protein [152]. Indeed, mutations in this gene have been associated with the osteoporotic phenotype in the osteogenesis imperfecta. A polymorphism located on intron 1 (transition guanine to thymidine) and affecting a binding site for transcription factor SP1 is of particular clinical interest. This polymorphism is considered a possible genetic risk factor for clinically important conditions of osteoporotic fractures [141]. Particularly, the SP1 polymorphism has been related to a reduction in bone mineral density [153] and to disc degradation in older women and men [152]. Similar polymorphic changes have been identified within the COL11A1 gene, located on chromosome 1p21.1. In particular, the T allele of COL11A1 C4603T polymorphism may increase Intervertebral Disk Disease (IVDD) susceptibility [154]. Both the COL11A1 gene and the COL11A2 gene encode one of the two alpha chains of type XI collagen [154]. Mio et al. studied the association of the type XI collagen genes, such as COL11A1, COL11A2, and COL2A1, with IVDD, and observed that the COL11A1 gene SNVs, rs1463035 and rs1337185, as well as the COL11A2 gene SNV, rs2076311, were associated with disc bulges [155,156]. While Yang et al. observed for the COL11A2 gene that the carriers of the A allele for rs2071025 and carriers of the C allele for rs986522 presented an increased risk of IVDD [157]. The COL1A2 gene lies on chromosome 7q22 and contains 52 exons [158]. Polymorphisms between the Eco RI, Pvu II, and Del38 sites in the COL1A2 gene have been previously correlated to osteoporosis [159]. Furthermore, variants, such as p.(Arg708Gln), p.(Gly247Cys), and p.(Gly193Ser), previously found in osteogenesis imperfecta and idiopathic osteoporosis, were detected in atypical femoral fractures and patients presenting fractures associated with low spinal BMD, respectively [160]. An additional gene involved in collagen production, COL2A1, also plays a primary role in skeletal development, bone resorption, and homeostasis. COL2A1 has a significant impact on cortical and trabecular bone mass, and, therefore, may influence skeletal architecture. In fact, COL2A1 glycine mutations p.Gly144Val and p.Gly267Asp cause the prototypical COL2A1-disease Stickler syndrome [161]. On the other hand, variation c.1946G > C (p.Gly649Ala) on the COL9A1 gene, involved in synthesizing type IX collagen and located on chromosome 6q13, has been correlated to ossification of the posterior longitudinal ligament [162]. While the COL9A1 promoter region can be transactivated by SOX9, rs73354570 of SOX9 has been significantly associated with postmenopausal osteoporosis [163].

The gene PLS3 encoding plastin 3 was recognized to be involved in X-linked osteoporosis [164]. Furthermore, early-onset osteoporosis has been characterized as a heterozygous mutation of the Wnt family member 1 (WNT1) gene, while autosomal-recessive loss-of-function mutations at c.1096G > A/p.V366 M of the LRP5 gene, encoding for a receptor within the Wnt pathway, were identified as responsible for osteoporosis-pseudoglioma syndrome [129,165]. Mutations and polymorphisms related to osteoporosis have also been identified at the low-density lipoprotein receptor-related protein 5 (LRP-5) gene [166], which maps to chromosome 11q12-q13 and at the Osteoprotegerin gene [167], which is a soluble protein receptor for RANKL. Moreover, the gla matrix protein (MGP) plays an important role in bone and vascular mineralization, as confirmed by a MGP-deficient murine model, where –138T > C, –7G > A, and Thr83Ala SNPs were associated with bone loss [168]. Similarly, rs2288377, rs35767, and rs2229765 polymorphisms within Insulin-like growth factor (IGF) genes, which are critical regulators for bone cell function [169,170,171], and T116G and G287T polymorphisms in exon 2, and A224T in exon 3 of BMP-2 gene, have been strongly associated with osteoporosis [172].

Additional genes whose mutations have been related to an increased risk of osteoporosis and fractures are Cytochrome P450 (CYP1A1) gene [173]. CYP1A1’s two variants, which occur in 19% of healthy individuals, have been related to osteoporosis [174] and Transforming growth factor beta (TGF-β) which presents polymorphisms. These can be divided into three classes according to their position: promoter, coding, and intronic polymorphisms. In particular, the promoter polymorphisms of TGFB1, i.e., C-1348T and G-1369A, may hamper gene expression. While coding polymorphisms T29C and C788T could affect the protein structure [175]. Finally, a rare polymorphism in intron 4 has been associated with reduced bone mineral density and osteoporotic fracture [141]. Osteocalcin, with a C- > T transition in the gene promoter has also been related to low bone mineral density [176]. Furthermore, a polymorphism at the restriction enzyme Hind III site in the promoter region was identified, and together with SNV, rs1543294, are likely important candidate sites involved in bone mineral density and osteoporosis [177]. The polymorphic gene Apolipoprotein E (ApoE), with three common alleles (ε2, ε3, ε4) coding for three isoforms (E2, E3, E4), might play a role in osteoporosis. Indeed, the ApoE4 variant may be important in determining spine bone mass and hip fractures in postmenopausal women [178]. Additional genes whose polymorphisms and mutations have been related to osteoporosis comprise: (i) Sclerostin (SOST), whose polymorphisms have been associated with some parameters of osteoporosis, such as B bone mineral density or risk fracture. In particular, the SRP 10565insGGA, which is located upstream of the SOST transcriptional site has been found to prompt a decrease in bone mineral density in older women [179]; (ii) Calcitonin receptor, whose coding polymorphism causes a proline-leucine substitution at codon 436 of the gene has been reported in patients with reduced bone mass [141]; (iii) Interleukin-1 receptor antagonist (IL-1RN), whose 86 base pair VNTR polymorphism in the second intron of the coding sequence has been related to early postmenopausal bone loss at the spine [180]; (iv) osteonectin gene, whose polymorphisms have been suggested to play a role in inherited osteoporosis susceptibility, such as the haplotype (1046C-1599G-1970T), which has been correlated to lower bone density [181]. 

### 5.2. Rickets and Osteomalacia

Rickets and osteomalacia are both caused by vitamin D, calcium, or phosphorus deficiencies. Rickets and osteomalacia usually affect children and adults/elderly, respectively, while both can be genetic or acquired. Rickets is a metabolic bone disorder that causes weak, soft bones in children, as a result of inadequate mineralization due to a prolonged deficiency of vitamin D, calcium, and/or phosphorus metabolism [182]. The most frequent cause of rickets is nutritional vitamin D deficiency, whereas about 13% of total rickets is due to genetic problems associated with the absorption of calcium and phosphorus [183]. The latter rickets category can be divided into two groups: (i) disorders of vitamin D biosynthesis and action, such as vitamin D-dependent rickets, and (ii) hereditary hypophosphatemic rickets [182]. Vitamin D-dependent rickets types 1 and 2 are autosomal recessive genetic diseases due to mutations in the renal 1-hydroxylase (CYP27B1) and vitamin D receptor (VDR) genes, respectively [184,185]. Digenic inheritance has been reported in a family with hereditary hypophosphataemic rickets with hypercalciuria (HHRH), which harbors heterozygous mutations of the SLC34A1 and SLC34A3 genes, encoding the renal sodiumphosphate co-transporters type 2a and 2c, respectively [129]. Autosomal dominant hypophosphataemic rickets is associated with mutations in the fibroblast growth factor (FGF) family, FGF23 [186]. Furthermore, X-linked hypophosphataemic (XLH) rickets results from mutations in a phosphate endopeptidase on the X chromosome (PHEX) gene [185]. Mutations in the PHEX gene cause increased levels of hormone fibroblast growth factor 23 (FGF23), leading to renal phosphate squandering and poor skeletal and dental mineralization in this illness [187]. In general, XLH is usually inherited as an X-linked dominant trait, although other familial patterns may occur [188]. 

Osteomalacia is among the most common osteometabolic diseases and has been described as one of the most disabling bone diseases of the elderly. It is more common in elderly females than elderly males [189]. Osteomalacia is caused by disorders that lead to decreased mineralization of bone [190]. It is associated with poor bone quality causing atraumatic fractures, pseudofractures, delayed fracture healing, and bone pain [191]. This disease is defined by a marked softening of the bones and is commonly caused by a lack of vitamin D [190]. Perhaps the rarest cause of osteomalacia is that caused by a neoplasm, so-called tumor-induced osteomalacia (TIO) [192]. X-linked hypophosphatemia (XLH) is a rare, lifelong disease caused by loss-of-function mutations in the PHEX gene, resulting in an excess of FGF23, which impairs renal phosphate reabsorption and suppresses the production of 1,25-dihydroxyvitamin D. This process results in chronic hypophosphatemia and persistent osteomalacia [191].

### 5.3. Juvenile Paget’s Disease

Juvenile Paget’s disease is a rare disorder affecting bone growth, which appears during infancy or early childhood [193]. This disorder causes abnormally large, misshapen bones which fracture easily, while its symptoms become more severe during the adolescent growth spurt when bones grow quickly compared to adulthood. Genetically, Juvenile Paget’s disease is an autosomal dominant monogenic disorder caused by mutations in a member of the TNF-receptor superfamily named TNFRSF11B [193]. TNFRSF11B is a gene involved in bone remodeling. Mutations within its coding sequence cause an abnormally fast bone remodeling rate starting in childhood, thereby leading to a larger, weaker, less organized new bone tissue than physiological bone. This abnormally fast bone remodeling underlies serious problems as bones are highly prone to fracture. 

### 5.4. Osteogenesis Imperfecta

Osteogenesis imperfecta is a condition characterized by bone fragility and extra-skeletal symptoms [194]. Reduced bone strength leads to fractures in atypical locations or low-trauma fractures, while extra-skeletal presentations may include dental anomalies, joint hypermobility, hearing loss, blue-gray sclera, etc. The condition is present at birth and develops in children with a family history of the disorder [194]. Different inheritance modes have been identified, comprising both autosomal dominant and recessive monogenic inheritance. Specifically, mutations in the two genes coding for collagen type I, COL1A1 and COL1A2, are the most common cause of osteogenesis imperfecta. In the past 10 years, defects in at least 17 other genes have been identified as responsible for osteogenesis imperfecta phenotype, with either dominant or recessive transmission [195].

### 5.5. Osteopetrosis

Osteopetrosis, also known as Marble bone disease or Albers–Schönberg disease, is a rare genetic, heritable condition that causes increased bone density [45]. Osteopetrosis may be caused by mutations in at least 10 genes. Genetically and clinically, osteopetrosis is very heterogeneous, therefore, accurate molecular classification is relevant for prognosis and treatment [196]. The disease progresses as the bones grow; the cavities of the marrow are filled with compact bone which results in a reduced amount of marrow, which in turn reduces the bone’s capacity to produce red blood cells. This can lead to severe anemia. Three forms of osteopetrosis can be distinguished based on the pattern of inheritance: (i) autosomal recessive, (ii) autosomal dominant, and (iii) X-linked. The first, which accounts for the most severe forms, is caused by biallelic mutations in TCIRG1, CLCN7, OSTM1, SNX10, and PLEKHM1 genes, encoding for proteins involved in the acidification of the resorption lacunae and/or in vesicular transport and loss-of-function mutations leading to osteoclast-rich osteopetrosis. Furthermore, mutations in RANKL and its receptor RANK are associated with osteoclast-poor autosomal recessive, where osteoclastogenesis is blocked [197,198]. The second osteopetrosis form can be type I or II; both differ in the presentation of clinical features and genetic mutations located in the LRP5 and CLCN7 genes, respectively. Type I derives from enhanced osteoblast activity due to reduced LRP5 affinity for the extracellular antagonists SOST and dikkopf-1 (DKK-1) and consequent increased Wnt canonical signaling [199], while the most common cause of type 2 is the presence of inactivating mutations in the chloride channel 7 (CLCN7) gene, which results in ineffective, osteoclast-mediated bone resorption [200]. The X-linked type of osteopetrosis results from mutations in the IKBKG gene, which encodes for NEMO, the regulatory subunit of the IKK (IkappaB kinase) complex, essential for NF-kappa B signaling [201].

### 5.6. Fibrous Dysplasia

Fibrous dysplasia (FD) is a rare metabolic bone disease resulting in weakened fracture and deformity-prone bones, where bone is replaced by structurally unsound fibro-osseous tissue [202]. FD is characterized by a highly disorganized mixture of immature fibrous tissue and fragments of immature trabecular bone [203]. It is an uncommon mosaic disorder caused by sporadic post-zygotic activating mutations in GNAS, resulting in dysregulated GαS-protein signaling in affected tissues [202]. Currently, there is no cure for FD, and treatment options may include surgery for relieving pain and repairing bones. FD presents along a broad clinical spectrum due to varying degrees of mosaicism, resulting in a disease that can range from asymptomatic to severely disabling [202]. Fibrous dysplasia may arise in isolation or as a multisystem developmental disorder known as McCune–Albright syndrome (MAS) [202,203]. Recent research suggests that the Wnt/B-catenin pathway may play a role in fibrous dysplasia, as patients with activating GNAS mutations specifically show that Gas mutations activated Wnt/B-catenin signaling [204].

### 5.7. Pyle Disease

Pyle disease is a rare autosomal recessive monogenic bone disorder [205]. Its main feature is the irregular development of long bones in the arms and legs. In this process, the trabecular bone is expanded, while cortical bone becomes thinner than normal. As a result, bones became fragile and are prone to fracture. Pyle disease is a monogenic disease that is inherited in an autosomal recessive pattern, where both copies of the secreted frizzled-related protein 4 gene (SFRP4) are mutated [205]. SFRP4 is involved in Wnt signaling. Regulation of Wnt signaling by the SFRP4 protein is critical for normal bone development and remodeling. The dysregulation of Wnt signaling due to SFRP4 mutation leads to the bone abnormalities characteristic of Pyle disease [205]. 

### 5.8. Additional Rare Metabolic Bone Diseases

Additional monogenic metabolic bone diseases include McCune–Albright syndrome (MAS) [206], which can be caused by sporadic postzygotic mosaicism, and Familial hypocalcemia (FHH), which is an autosomal dominant condition presenting mutations in the calcium-sensing receptor (CaS receptor) signaling pathway [207]. FHH is also associated with mutations on GNA11, A25S1, and CASR genes [208]. FHH causes hypercalcemia by three genetic mechanisms: (i) inactivating mutations in the calcium-sensing receptor, (ii) the G-protein subunit α11, (iii) adaptor-related protein complex 2, sigma 1 subunit [207]. 

The monogenic bone disorder Autosomal dominant hypocalcemia (ADH) type 1 is caused by heterozygous activating mutations in the calcium-sensing receptor (CASR), which increase the CASR sensitivity to extracellular ionized calcium [209]. Extracellular calcium is essential for life and its concentration in the blood is maintained within a narrow range. This is achieved by a feedback loop that receives input from CASR, expressed on the surface of parathyroid cells. In response to low ionized calcium, the parathyroids increase secretion of parathyroid hormone (PTH), which increases circulating calcium levels [209]. CASR is also highly expressed in the kidneys, where it regulates calcium reabsorption from the primary filtrate. 

Mutations on GNAS, NESP55, and STX16 genes are associated with pseudohypoparathyroidism (PHP), a rare disease inherited in an autosomal dominant pattern. PHP is marked by parathyroid (PTH) resistance, hypocalcemia, and hyperphosphatemia caused by a GNAS (guanine nucleotide-binding protein-subunit) mutation (PHP1A) or epimutation (PHP1B) [210].

Additional genetic pathologies affecting bone tissue comprise sclerosteosis (SOST) types 1 and 2, which are inherited in an autosomal recessive pattern. Sclerosteosis type 1 is caused by ten homozygous loss-of-function mutations within the gene SOST that encodes the inhibitor of Wnt-mediated bone formation, sclerostin. Sclerosteosis type 2 is a condition caused by one heterozygous or two homozygous loss-of-function mutations in the lipoprotein receptor-related protein 4 (LRP4) gene, which is involved in bone homeostasis [211]. 

## 6. Conclusions

In conclusion, different signaling pathways, including TGF-β/BMP and Wnt/β-catenin pathways, as well as epigenetic processes, including DNA methylation, histone post-translational modifications, miRNAs, lnc-RNAs, and circRNAs, play a pivotal role in bone formation and turnover. The identification of specific epigenetic markers could be extremely useful for assessing individuals at risk of future non-communicable disease and allowing novel pathways that influence the phenotype to be discovered. Detecting such indicators early in life, even in peripheral tissues, could provide useful predictive markers for the later phenotype in cell types that are more relevant to different bone metabolic diseases, thus allowing better treatment options to be considered. Moreover, epigenetics may be a potential target for the treatment of bone diseases. 

Mutations and dysfunctions in pathways that regulate bone turnover might influence the bone remodeling process, ultimately leading to a large variety of metabolic bone disorders. A large fraction of metabolic bone diseases presents a genetic basis and represents either a (i) monogenic disorder due to a germline or somatic single gene mutation, or a (ii) digenic, oligogenic, and polygenic disorder that involves variants in more than one gene. Identifying these heritable diseases represents a significant clinical opportunity, as it can enable early recognition and therefore widen therapeutic options. High throughput methods might strongly improve the identification of genetic abnormalities linked to metabolic bone diseases. Therefore, it is of paramount importance to develop new strategies aimed at pinpointing the genetic background of these diseases, in order to improve patient outcomes and to produce novel therapies. Further studies in this direction are urgently needed. 

## Data Availability

Not applicable.
